# Systemic impacts of metabolic dysfunction-associated steatotic liver disease (MASLD) and metabolic dysfunction-associated steatohepatitis (MASH) on heart, muscle, and kidney related diseases

**DOI:** 10.3389/fcell.2024.1433857

**Published:** 2024-07-16

**Authors:** Reddemma Sandireddy, Suganya Sakthivel, Priyanka Gupta, Jatin Behari, Madhulika Tripathi, Brijesh Kumar Singh

**Affiliations:** Cardiovascular and Metabolic Disorders Research Program, Duke-NUS Medical School, Singapore, Singapore

**Keywords:** Adipose tissue (AT), metabolic dysfunction-associated steatotic liver disease (MASLD)/non-alcoholic fatty liver disease (NAFLD), metabolic dysfunction-associated steatohepatitis (MASH)/non-alcoholic steatohepatitis (NASH), cardiovascular diseases (CVDs), sarcopenia, chronic kidney diseases (CKDs), heart, muscle

## Abstract

Metabolic dysfunction-associated steatotic liver disease (MASLD), previously known as non-alcoholic fatty liver disease (NAFLD), is the most common liver disorder worldwide, with an estimated global prevalence of more than 31%. Metabolic dysfunction-associated steatohepatitis (MASH), formerly known as non-alcoholic steatohepatitis (NASH), is a progressive form of MASLD characterized by hepatic steatosis, inflammation, and fibrosis. This review aims to provide a comprehensive analysis of the extrahepatic manifestations of MASH, focusing on chronic diseases related to the cardiovascular, muscular, and renal systems. A systematic review of published studies and literature was conducted to summarize the findings related to the systemic impacts of MASLD and MASH. The review focused on the association of MASLD and MASH with metabolic comorbidities, cardiovascular mortality, sarcopenia, and chronic kidney disease. Mechanistic insights into the concept of lipotoxic inflammatory “spill over” from the MASH-affected liver were also explored. MASLD and MASH are highly associated (50%–80%) with other metabolic comorbidities such as impaired insulin response, type 2 diabetes, dyslipidemia, hypertriglyceridemia, and hypertension. Furthermore, more than 90% of obese patients with type 2 diabetes have MASH. Data suggest that in middle-aged individuals (especially those aged 45–54), MASLD is an independent risk factor for cardiovascular mortality, sarcopenia, and chronic kidney disease. The concept of lipotoxic inflammatory “spill over” from the MASH-affected liver plays a crucial role in mediating the systemic pathological effects observed. Understanding the multifaceted impact of MASH on the heart, muscle, and kidney is crucial for early detection and risk stratification. This knowledge is also timely for implementing comprehensive disease management strategies addressing multi-organ involvement in MASH pathogenesis.

## Introduction

Recently, the Delphi process, led by an international consortium of pan-liver associations, has critically evaluated the terminological shortcomings associated with “non-alcoholic fatty liver disease (NAFLD).” As a result, the term has been redefined as “metabolic dysfunction-associated steatotic liver disease (MASLD)” (Rinella et al., 2023). This non-stigmatizing updated terminology, coupled with enhanced diagnostic guidelines, will support both awareness and the accuracy of patient identification, surpassing the limitations of previous nomenclature NAFLD. However, the inflammatory phase of MASLD, steatohepatitis, has been identified as a critical pathophysiological entity, deserving preservation of the term “steatohepatitis” within the clinical context. Consequently, it has been recommended that this condition be reclassified as “metabolic dysfunction-associated steatohepatitis (MASH),” formerly recognized as non-alcoholic steatohepatitis (NASH) (Estes et al., 2018; Fan XZ et al., 2024). MASH represent a spectrum of liver conditions consisting hepatic inflammation and fibrosis associated to metabolic dysfunction with the absence of significant alcohol consumption (Lim et al., 2023; Cusi et al., 2024; Fan XZ et al., 2024). These conditions have emerged as the leading causes of liver-related morbidity and mortality globally (Younossi et al., 2024a; Younossi et al., 2024b). MASLD/MASH is characterized by excessive hepatic fat accumulation, hepatic inflammation, hepatocyte ballooning and fibrosis, which may progress to cirrhosis and hepatocellular carcinoma (HCC) (Sinha et al., 2018; Tripathi et al., 2022).

The history of clinical MASH/NASH studies began about 45 years ago (Adler and Schaffner, 1979; Ludwig et al., 1980), but its recent increase in prevalence poses it as a global pandemic (Pouwels et al., 2022). Several recent studies have quantified the overall global prevalence of MASLD to be 30%–33%. Notably, reports show an increasing rate of MASLD prevalence from 25.3% (1990–2006) to 38.2% (2016–2019), which is a 50.4% increase in the last 30 years (Le et al., 2023). NAFLD/MASLD prevalence increased from 35.42% (2008–2016) to 46.20% (2017–2020) in MENA (Middle East and North Africa) region (Younossi et al., 2024b). Currently, the progression from MASLD to cirrhosis is estimated to be 4%. However, 20% of MASH patients could progress to cirrhosis. Most importantly, recent reports showed a dramatic increase in liver transplant waitlist registration accounted for MASH and associated cirrhosis and HCC (Lim et al., 2023; Younossi et al., 2024c). Epidemiologically, MASH possesses high geographic variability with higher rates encountered in South America and the Middle East, followed by Asia (Younossi et al., 2023). However, a higher MASLD incidence is recorded in the younger (45 years or younger) Asian population, which could be attributed to carbohydrate-rich foods, high central adiposity, and genetic predisposition (Younossi et al., 2023). Africa has recorded a low rate of MASLD (Younossi et al., 2023). Moreover, MASLD and MASH are highly associated (50%–80%) with impaired insulin response, type 2 diabetes, dyslipidaemia, hypertriglyceridemia, hypertension, and more than 90% of obese patients with type 2 diabetes have MASH (Le et al., 2023). Considering its significant association with other metabolic comorbidities, modelling study by Estes et al. (2018), has forecasted the prevalent MASLD cases to increase by 21% from 2015 to 2030, while prevalent MASH cases will increase by 63%. A following study by Estes et al. suggested a potential increase of 6%–20% in the prevalence of MASLD cases across Hong Kong, Singapore, South Korea, and Taiwan between 2019 and 2030. Similarly, they projected a concurrent rise of 20%–35% in prevalent MASH cases during the same period (Estes et al., 2020). Furthermore, their forecast anticipated a substantial surge in MASLD-related mortality, with estimates ranging between 65% and 100% from 2019 to 2030, prompting a significant concern (Estes et al., 2020).

Scientists, thus, predict that MASH could become the top condition for liver transplants soon. Despite its growing prevalence rate, to date, there are no specific therapies approved by the US Food and Drug Administration for MASLD/MASH (Tripathi et al., 2022). Treatment strategies for MASH typically involve lifestyle interventions, such as adherence to Mediterranean diet and increased physical activity (Semmler et al., 2021; Pouwels et al., 2022). However, for patients who do not respond to these interventions, several drugs targeting FXR, PPAR and GLP-1R agonists, focusing on inflammation, ballooning, apoptosis, and fibrosis, are currently in development (Jensen et al., 2018; Loomba et al., 2021; Duan et al., 2022). Bariatric surgery is designated for individuals classified as morbidly obese who have shown inadequate response to lifestyle interventions or weight-loss medications (Nachit et al., 2021; Pouwels et al., 2022). Studies indicated that direct medical expenses for MASH could sum up to $222 billion (excluding indirect and societal costs) (Witkowski et al., 2022).

**FIGURE 1:**
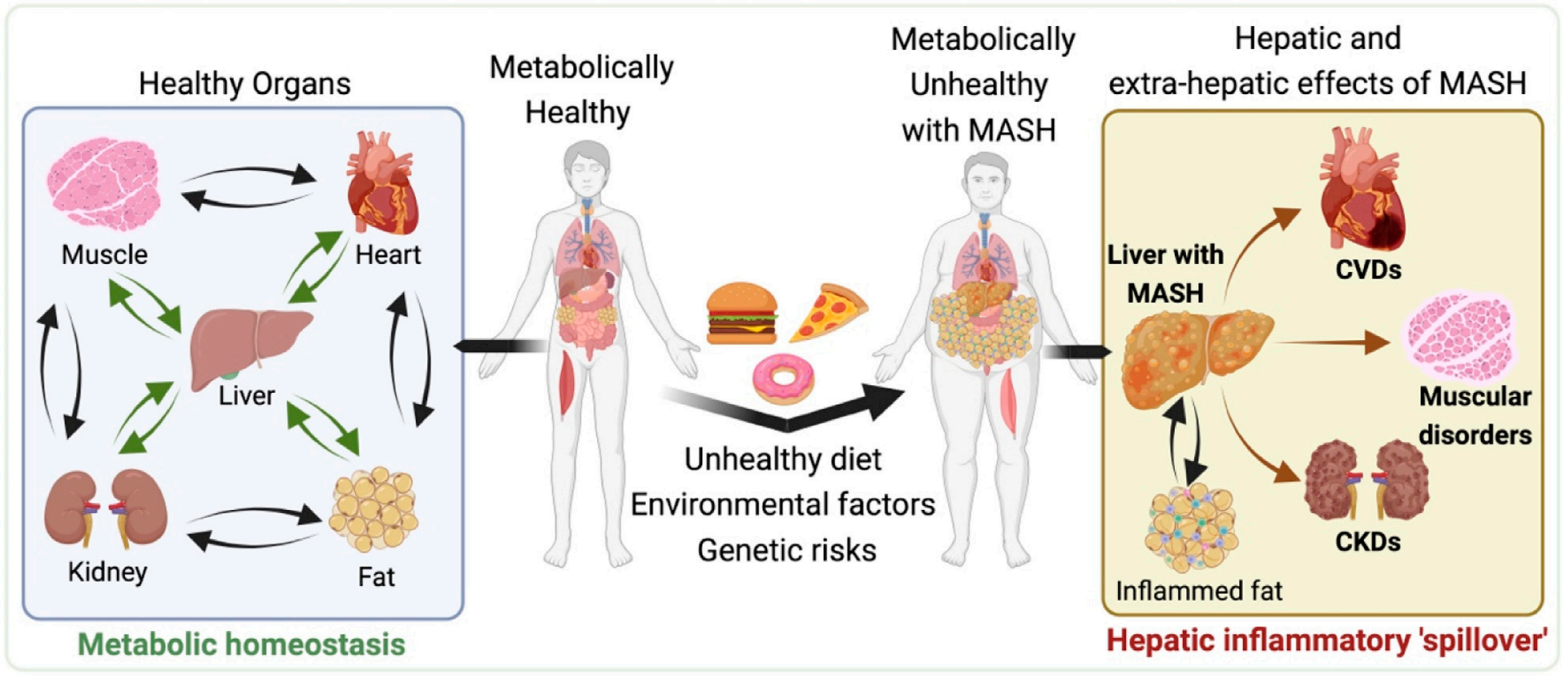
Systemic Impacts of metabolic dysfunction-associated steatotic liver disease (MASLD) and metabolic dysfunction associated steatohepatitis (MASH). Illustration depicts the progression from a metabolically healthy state (left side) to the development of MASLD and MASH and its subsequent systemic effects (right side). The left side shows the interconnectedness of healthy organs, including the heart, liver, muscle, and kidneys, maintaining metabolic balance. However, moving to the right, the impact of an unhealthy diet, environmental factors, and genetic predispositions contribute to the development of MASH, characterized by enlarged liver and visceral fat depot with the accumulated fat and inflammation. The hepatic and extra-hepatic consequences of MASH are evident, as the liver afflicted with MASH causally associated with the increased risk of cardiovascular diseases (CVDs), muscular disorders and chronic kidneys diseases (CKDs). All of which may also adversely impacted by the inflamed visceral fat associated with MASH. This representation highlights the systemic pathological nature of MASH, highlighting the importance of a holistic approach to its management and treatment. The illustration was made on BioRender.com.

MASLD and MASH have long been recognized primarily as a liver disease, but recent insights have expanded our understanding to acknowledge its significant systemic implications, especially concerning the cardiovascular, muscular, and renal systems (Nachit et al., 2021; Li et al., 2023; Wegermann et al., 2023). These organs are selected for focused study due to their vital roles in metabolic regulation and their pronounced vulnerability to the metabolic derangements typically associated with MASH, such as insulin resistance (IR), dyslipidemia, and systemic hypertension (Ferrara et al., 2019; Kasper et al., 2021). The pathological processes of MASH, including inflammation and fibrosis, exert profound systemic effects that are particularly observable in these organs (Chakravarthy et al., 2020; Wang et al., 2022). These effects include cardiovascular dysfunction, muscle wasting, and renal impairment, which not only exacerbate the disease burden but also critically influence patient outcomes by contributing significantly to morbidity and mortality (Ferrara et al., 2019; Wang et al., 2022), as illustrated in Figure 1. In contrast, while MASH undoubtedly impacts other tissues, the effects on these non-core systems are often less direct and may not significantly alter disease prognosis or treatment strategies. For instance, the gastrointestinal tract, skin, or adipose tissue (AT) might experience alterations due to metabolic dysfunction; however, these changes may not typically result in immediate life-threatening consequences or require the urgent, targeted interventions demanded by cardiac, muscular, or renal involvement (Alonso-Pena et al., 2023; Zeng et al., 2024). Furthermore, while this review primarily addresses the direct impacts and interactions of liver with heart, kidney and muscle in MASLD/MASH, the roles of other tissues, notably AT, in influencing systemic health outcomes are equally pivotal but beyond the scope of this manuscript. AT, through its endocrine and paracrine functions, significantly affects the heart, muscles, and kidneys, primarily via the secretion of adipokines that modulate inflammation, insulin sensitivity, and lipid metabolism. These interactions are complex and contribute to the multisystem nature of metabolic disorders. For a detailed exploration of the systemic effects of AT and its implications for cardiovascular, musculoskeletal, and renal health in the context of MASLD/MASH, readers are encouraged to consult the following comprehensive reviews (Chow et al., 2022; Gilani et al., 2024; Hafiane, 2024; Jia et al., 2024). Therefore, this manuscript prioritizes the heart, muscles, and kidneys due to their critical interplay with metabolic health and their direct connection to the primary complications associated with MASH, thereby offering clearer targets for therapeutic intervention and management strategies.

Systemic manifestations, as indicated above, are largely attributed to the metabolic dysregulation underlying MASLD/MASH, including obesity, IR, systemic inflammation, and lipid dysmetabolism, which play pivotal roles in the pathogenesis of cardiovascular, muscular and renal complications (Anstee et al., 2020; Venniyoor et al., 2021). The systemic nature of MASLD/MASH and its extrahepatic manifestations necessitates a multidisciplinary approach for its management, integrating the expertise of hepatologists, endocrinologists, cardiologists, nephrologists, and nutritionists. Furthermore, this complexity highlights the importance of ongoing research to unravel the pathophysiological mechanisms linking MASH with its systemic effects, aiming to identify novel therapeutic targets and improve patient outcomes. Thus, the advancements in understanding of MASLD/MASH as a multisystem disease highlights the urgent need for heightened awareness and comprehensive management strategies. This review aims to consolidate present understanding regarding the hepatic and extrahepatic presentations of MASH, which contribute to cardiovascular, muscular, and renal complications, thereby laying the groundwork for further advancements in research and clinical practices.

## Liver complications of MASLD/MASH

The hepatic manifestations of MASH involve a complex interplay of metabolic dysfunction and liver pathology. The pathogenesis of MASH is elucidated through the “multiple hit hypothesis,” which supersedes the earlier simplistic “two-hit model” by integrating multiple metabolic, genetic, epigenetic, and environmental factors that collectively drive disease progression. Over the past two decades, research has suggested that the initiation of MASH occurs with the accumulation of excessive intrahepatic fat, exceeding 5% of the total liver weight (Rinella et al., 2023). This accumulation then triggers metabolic disturbances, including alterations in pathways associated with fatty acid oxidation, dysregulated signaling of reactive oxygen species (ROS), mitochondrial dysfunction, compromised proteostasis, and imbalances in gut microbiome (Buzzetti et al., 2016; Machado and Diehl, 2016; Mitten and Baffy, 2022; Rafaqat et al., 2024; Zhong et al., 2024).

**FIGURE 2:**
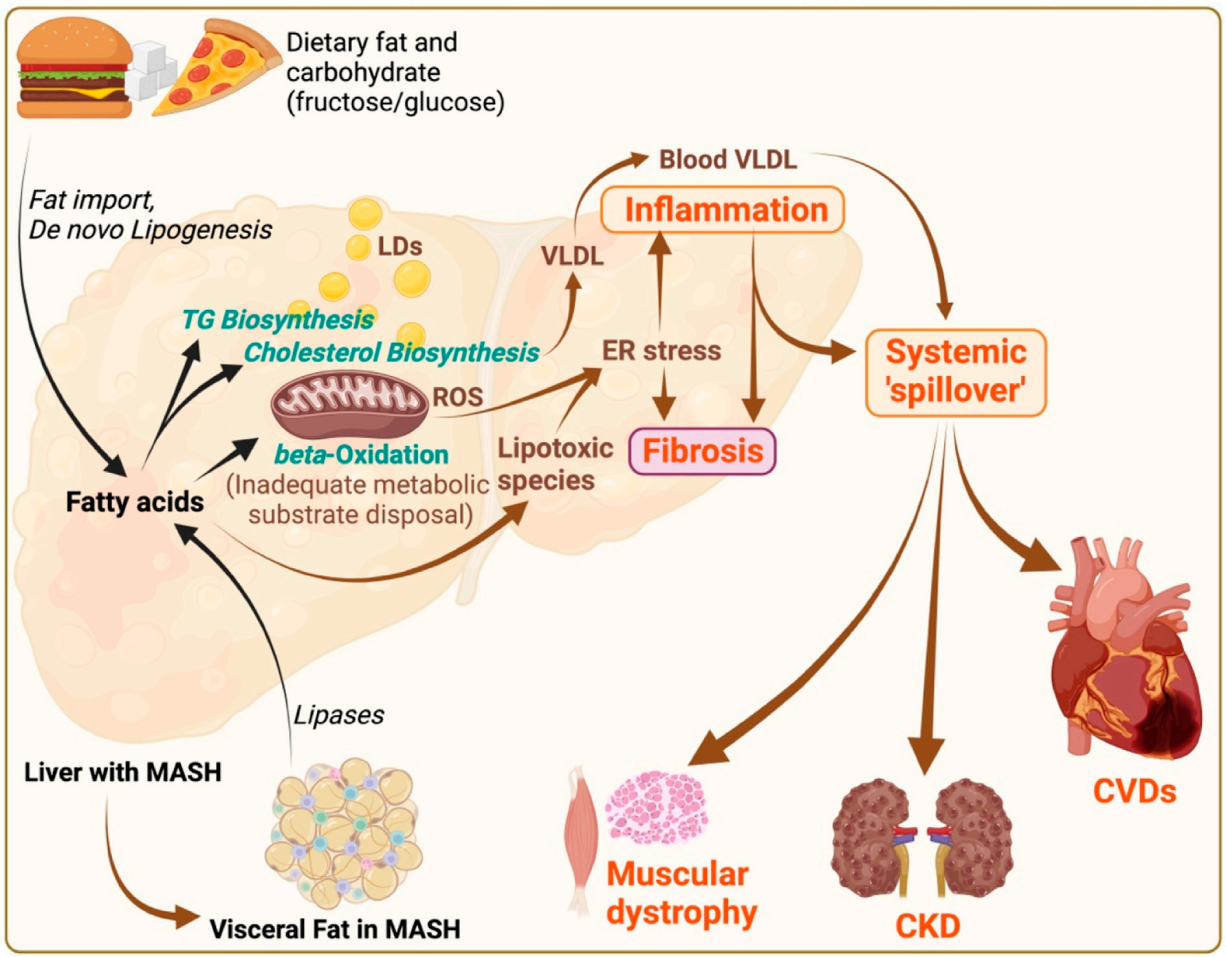
MASLD/MASH pathogenesis and its systemic implications. Illustration depicts the pathophysiological mechanisms of metabolic dysfunction-associated steatotic liver disease (MASLD)/metabolic associated steatohepatitis (MASH), and its systemic consequences. Dietary fats and carbohydrates (fructose in particular) contribute to fat import and *de novo* lipogenesis respectively within the liver, leading to triglyceride (TG) and cholesterol biosynthesis, and subsequent fatty acid accumulation. Increased flux of free fatty acids to mitochondria causes mitochondrial burnout, leading to inadequate disposal of metabolic substrates due to impaired beta-oxidation. These processes result in; i) increased lipid droplets (LDs) causing steatosis, ii) increased very-low-density lipoprotein (VLDL) production, iii) oxidative stress marked by reactive oxygen species (ROS), and iv) endoplasmic reticulum (ER) stress, promoting lipotoxicity deriving hepatic inflammation and fibrosis. The systemic “spill over” of inflammation from the liver impacts other organs, including the heart, muscles, and kidneys. This “spill over” leads to extrahepatic pathologies such as cardiovascular diseases (CVDs), muscular dystrophy, and chronic kidney disease (CKD). Additionally, visceral fat (with inflammation and fibrosis) in MASH, characterized by increased lipases activity, contributes to the systemic inflammatory responses, thereby exacerbating the cycle of metabolic dysfunction across multiple organ systems. The illustration was made on BioRender.com.

The implications of increased free FA accumulation involve augmented β-oxidation rates (referred as “inadequate substrate disposal”) and escalated ROS production within the mitochondrial respiratory chain (Figure 2). This imbalance between ROS generation and antioxidant defence mechanisms instigates oxidative stress, a hallmark of MASH pathogenesis, corroborated by elevated oxidative stress biomarkers in affected individuals. The oxidative milieu activates endoplasmic reticulum (ER) stress facilitating the activation of hepatic immune cells, including hepatocytes, hepatic stellate cells, Kupffer cells, dendritic cells, natural killer cells, T-lymphocytes, and B-lymphocytes, alongside pro-inflammatory signalling pathways. Free FAs exacerbate this inflammatory cascade, fostering the secretion of pro-inflammatory cytokines such as interleukin-6 (IL-6), tumor necrosis factor α (TNFα), and interleukin-1β (IL-1β), thereby perpetuating hepatic inflammation and cellular injury. These molecular derangements compromise the liver’s capacity for storing and exporting free FAs as triglycerides, culminating in hepatocyte lipid overload, steatosis and lipotoxicity. These mechanisms are comprehensively reviewed elsewhere (Buzzetti et al., 2016; Machado and Diehl, 2016; Lebeaupin et al., 2018; Loomba et al., 2021; Powell et al., 2021; Tilg et al., 2021; Llovet et al., 2023; Fan XZ et al., 2024; Habibullah et al., 2024; Jiang et al., 2024; Mahmoudi et al., 2024; Rafaqat et al., 2024; Targher et al., 2024; Verma MKT et al., 2024; Zhang et al., 2024; Zhong et al., 2024).

Adding to this hepatic injury, dysfunction of AT/fat deposits during obesity and type 2 diabetes mellitus (T2D) amplifies circulating free FAs and pro-inflammatory cytokines, intensifying the MASH progression and *vice versa*. This dysregulation perhaps extends beyond hepatic boundaries, affecting systemic metabolic processes such as glucose uptake, glycogen synthesis, glucose and fat oxidation, lipid storage, and lipolysis. Ectopic fat deposition with increased inflammation resulting from MASLD and obesity in non-AT, pose a substantial risk factor for CVDs, highlighting the severe pathogenic impact of MASH beyond the liver (Ferrara et al., 2019; Njoku et al., 2022). Defects in hepatic lipid metabolism not only precipitate intrahepatic lipid accumulation but also promotes lipid storage in non-hepatic tissues, highlighting the liver’s central role in systemic lipid homeostasis. Altered hepatic lipid metabolism under MASH conditions further predisposes individuals to atherogenic dyslipidaemia, characterized by elevated low-density lipoprotein (LDL) cholesterol levels and the ensuing formation of atherosclerotic plaques within arterial walls (Huang et al., 2023; Zhu et al., 2023). Thus, MASH is increasingly recognized not merely as a liver-specific ailment but as a systemic disorder with far-reaching effects on multiple organs. This condition is intricately linked with an elevated risk of a spectrum of severe health outcomes, including both fatal and non-fatal CVDs (64% higher risk), T2D, sarcopenia, and CKDs (Peng et al., 2022; Kawaguchi et al., 2023). The underlying pathophysiology connecting MASH to these systemic complications is multifaceted, involving metabolic dysregulation, inflammation, and altered lipid homeostasis.

The precise mechanisms underlie the causal relationship between MASH and extrahepatic diseases is not fully elucidated. However, it is likely that systemic inflammation and spill over mechanisms play a role in promoting inflammation in other tissues. Central to the systemic nature of MASH is the concept of hepatic inflammatory “spill over,” where pro-inflammatory mediators produced in the liver (Njoku et al., 2022), as a consequence of lipid accumulation and cell injury (lipotoxicity), disseminate through the bloodstream to distant organs (Buzzetti et al., 2016; Loomba et al., 2021; Zhong et al., 2024). This process is facilitated by an array of cytokines, chemokines, and other inflammatory molecules, such as TNF-α, interleukins (e.g., IL-6, IL-1β), and C-reactive protein (CRP), which are elevated in the context of MASH (Buzzetti et al., 2016; Machado and Diehl, 2016; Mitten and Baffy, 2022). These mediators can exacerbate pathological processes in the heart, skeletal muscle, and kidneys, contributing to the multisystem impact of the disease (Hong et al., 2014; Buzzetti et al., 2016; Machado and Diehl, 2016; Bhanji et al., 2017a; Mitten and Baffy, 2022). In this review, we inspect both pre-clinical and clinical evidence to refine our understanding of MASH and its wide-reaching pathological effects on critical organs such as the heart, muscles, and kidneys. A comprehensive, multi-dimensional therapeutic strategy is advocated to improve liver injury while also mitigating adverse cardiovascular and metabolic consequences. This highlights the importance of adopting a holistic/integrated management approach to address the systemic manifestations of MASH.

## Cardiovascular complications in MASLD/MASH

MASLD and MASH are increasingly recognized for its systemic implications that exceeds hepatic boundaries. The pathophysiological nexus between MASH and a constellation of extrahepatic conditions, notably CVDs, manifests as a multifaceted challenge in clinical management (Ferrara et al., 2019; Zhu et al., 2023). CVDs are the predominant cause of mortality in patients with MASH, outpacing liver-centric complications and malignancies (Le et al., 2023; Choe et al., 2024). This complex relationship between MASH and cardiovascular morbidity is underscored by shared and interlinked risk factors, including but not limited to IR, T2D, hypertension, dyslipidaemia, metabolic syndrome, and notably, liver fibrosis (Houghton et al., 2019; Ismaiel and Dumitrascu, 2019). Furthermore, the relationship between the likelihood of developing cardiovascular abnormalities and the severity of hepatic fibrosis in MASH is strongly correlated (Yang et al., 2022; Wegermann et al., 2023).

The spectrum of cardiovascular complications associated with MASH is broad, encompassing coronary artery diseases (CAD), atherosclerosis (ASCVD), cardiac remodelling anomalies, hypertrophy, heart failure, and arrhythmias. The precise mechanisms by which MASH contributes to cardiovascular pathology are not fully understood. However, emerging evidence suggests that hepatic fat accumulation serves as a predictor for impaired myocardial metabolism and subsequent cardiac dysfunction (Ferrara et al., 2019; Lim et al., 2019; Njoku et al., 2022; Younossi et al., 2024a; Wang et al., 2024). Furthermore, MASLD is strongly associated with structural and functional cardiac abnormalities, such as left ventricular hypertrophy, enhanced epicardial fat thickness, and various arrhythmogenic manifestations, likely stemming from the liver’s pivotal role in systemic glucose and lipid homeostasis (Ismaiel and Dumitrascu, 2019; Patel and Siddiqui, 2019; Mantovani et al., 2022).

### MASH-associated atherosclerotic risk

Independent of traditional cardiovascular risk factors, MASLD/MASH constitutes a significant risk factor for atherosclerotic cardiovascular diseases (ASCVD) (Pais et al., 2019; Arslan and Yenercag, 2020). The onset of MASH is correlated with an elevated production and release of LDL and VLDL particles from the liver during hepatic steatosis. These circulating LDL and VLDL particles in blood then accumulate beneath the arterial lining, leading to the formation of atherosclerotic plaque (Siddiqui et al., 2015; Sinha et al., 2018). Increasingly, research studies have revealed a notable correlation between hepatic steatosis and an augmented vulnerability to subclinical atherosclerosis (Yan et al., 2023; Zhu et al., 2023). Moreover, liver’s exacerbated secretion of pro-inflammatory cytokines, vasoactive substances, and pro-coagulant molecules further scaffolds the cardiovascular disease architecture in MASH patients (Garbuzenko, 2022; Huang et al., 2022).

More commonly, MASH is closely linked to the elevated levels and interactions of various cytokines and chemokines, which orchestrate an inflammatory response that directly impairs endothelial dysfunction during atherosclerosis. Among the key players, Tumor Necrosis Factor-alpha (TNF-α) and Interleukin-6 (IL-6) are central to this process. TNF-α exacerbates endothelial dysfunction by inhibiting nitric oxide (NO) synthesis, a crucial regulator of vascular tone and endothelial health, and by inducing the expression of vascular cell adhesion molecules that promote leukocyte adherence and vascular inflammation (Dimitroglou et al., 2023). IL-6, while possessing both pro- and anti-inflammatory properties, in the context of MASH tends to promote inflammation, contributing to vascular permeability and endothelial activation, thereby facilitating atherosclerosis (Zegeye et al., 2018). Further compounding the endothelial response are Interleukin-1β (IL-1β) and Interleukin-18 (IL-18), which potentiate the inflammatory milieu and are implicated in the upregulation of adhesion molecules and the recruitment of inflammatory cells to endothelial sites, promoting plaque formation and vascular stiffness (Krishnan et al., 2014). Additionally, C-reactive protein (CRP), an acute-phase reactant upregulated in response to IL-6, indirectly influences endothelial function by decreasing NO availability and promoting endothelin-1 production, a potent endothelial activator and vasoconstrictor (Pasceri et al., 2000). Furthermore, monocyte chemoattractant proteins (MCPs), particularly MCP-1, and the C-X-C motif chemokine ligands (CXCLs) like CXCL8 and CXCL12, also play pivotal roles. MCP-1 recruits monocytes to the endothelium, facilitating their transformation into macrophages and foam cells, a key step in atherogenesis (Medrano-Bosch et al., 2023; Su et al., 2023). CXCL8 (IL-8), known for its potent chemotactic abilities, further recruits neutrophils and exacerbates local inflammation, while CXCL12 is involved in stem cell recruitment and tissue repair, highlighting a complex balance of detrimental and potentially reparative mechanisms within the inflamed vasculature (Schober, 2008). Thus, elucidating these molecular interactions offers crucial insights into the pathophysiological mechanisms underlying endothelial dysfunction in MASH. Additionally, quantifying these cytokines may serve as biomarkers for assessing the increased risk of atherosclerotic cardiovascular diseases (ASCVD) associated with MASH.

Moreover, carotid intima-media thickness (CIMT), a non-invasive ultrasound measure of the combined thicknesses of the intimal and medial layers of the carotid artery wall, stands as a validated biomarker for subclinical atherosclerosis (Ismaiel and Dumitrascu, 2019; Ren et al., 2023). A substantial correlation between CIMT and the severity of MASH has been documented, implicating hepatic pathology in MASH as a potential precursor of heightened cardiovascular risk (Ismaiel and Dumitrascu, 2019; Ren et al., 2023). This observed association extends to the severity of histological features within MASLD and MASH, with more pronounced CIMT corresponding to increasingly severe liver pathology (Targher et al., 2006; Nahandi et al., 2014). This implies a potential bidirectional influence, wherein systemic inflammation and metabolic dysregulation inherent to MASH exacerbate vascular pathology and *vice versa*.

Furthermore, laboratory markers such as gamma-Glutamyltransferase (GGT) and alanine aminotransferase (ALT) have emerged as potential indicators of increased CIMT in MASH patients, highlighting the systemic nature of the disease and its impact on vascular health (Targher et al., 2006). Despite these insights, the pathophysiological mechanisms underpinning the relationship between MASH and ASCVD remains unclear. Factors such as the direct impact of hepatic steatosis on endothelial function, the role of liver-derived proinflammatory cytokines in vascular inflammation, and the contribution of metabolic dysregulation to atherogenesis are areas of ongoing investigation (VanWagner, 2018; Muzurovic et al., 2022). Further, the relationship between MASH and carotid artery disease is becoming more substantiated by a growing body of scientific/clinical evidence (Pais et al., 2019; Huang et al., 2022). Carotid artery disease, characterized by the narrowing or blockage of the carotid arteries which are pivotal for cerebral blood supply (Ratchford and Evans, 2014), has been observed with greater prevalence among individuals diagnosed with MASLD and MASH (Simons et al., 2022; Tang et al., 2022). This association between MASH and carotid artery disease involves various risk factors such as elevated body mass index (BMI), active smoking, elevated levels of LDL, IR, and the presence of metabolic syndrome, contributing to carotid artery disease pathogenesis in MASH (Peng et al., 2020; Katsiki et al., 2021; Zhao J. et al., 2022; Ren et al., 2023). Thus, strategies for accurately evaluating atherosclerosis risk in MASH patients are yet to be established.

### MASH-associated coronary microvascular dysfunction

Coronary microvascular dysfunction represents a critical facet of cardiovascular pathology in patients with MASLD and MASH, delineating a spectrum of abnormalities that include impaired endothelial function, reduced coronary artery flow reserve, and compromised collateral vessel formation in response to ischemia (Villanova et al., 2005). Coronary microvascular dysfunction in MASH encompasses a pattern of detrimental effects on the coronary microcirculation, not limited to the endothelial layer but extending to the smooth muscle cells that regulate vascular tone and, consequently, myocardial blood flow and ultimately resulting in myocardial ischemia (Camici and Crea, 2007; Yang et al., 2023). Impaired endothelial function, a hallmark of this dysfunction, results from endothelial cells’ reduced capacity to produce NO, a potent vasodilator, in response to stimuli which required to prevent ischemic injury (Yang et al., 2023). This impairment is closely linked to the systemic inflammatory state and IR inherent to MASH, which also predispose to the formation of coronary plaques that are particularly prone to rupture, further exacerbating the risk of acute coronary events (Del et al., 2021).

Coronary flow reserve (CFR), the ratio of maximal flow to resting flow in the coronary circulation, emerges as a pivotal measure in this context. A reduced CFR signifies an inability to sufficiently increase blood flow to meet myocardial demands during stress, indicating both epicardial stenosis and microvascular myocardial perfusion abnormalities (Villanova et al., 2005; Murthy et al., 2011). Advanced imaging techniques, such as positron emission tomography/computed tomography (PET/CT) scans, enable the quantitative assessment of myocardial perfusion imaging (MPI), offering an integrated view of the heart’s vascular function (Murthy et al., 2011). This approach underscores the systemic impact of MASH on cardiovascular health by providing insights into the compromised myocardial perfusion characteristic of coronary microvascular dysfunction. The presence of both coronary microvascular and diastolic dysfunctions has been associated with an increased risk of developing heart failure with preserved ejection fraction (HFpEF) events (Taqueti et al., 2018).

### MASH-associated risk of heart failure with preserved ejection fraction

The complex link between coronary microvascular dysfunction and HFpEF development in MASH patients is of growing interest. HFpEF, featuring heart failure symptoms alongside normal ejection fraction, is increasingly common in those with metabolic syndrome and MASH, primarily due to diastolic dysfunction and altered filling pressures (Yang et al., 2022; Wegermann et al., 2023). This condition constitutes a significant portion of heart failure cases, notably in MASH-related metabolic derangements. Clinical studies reveal pathophysiological pathways connecting MASH to HFpEF, emphasizing systemic inflammation, endothelial dysfunction, and myocardial fibrosis, leading to impaired heart muscle relaxation and elevated filling pressures, hallmarks of diastolic dysfunction. Paulus and Tschöpe (2013) propose an inflammatory HFpEF model, where comorbidities like MASH trigger systemic microvascular endothelial inflammation, resulting in coronary microvascular dysfunction, myocardial stiffness from fibrosis, and cardiomyocyte remodelling. Mohammed et al. (2014) demonstrate significant diastolic dysfunction in metabolic syndrome patients, similar to those with MASH, correlating with metabolic derangement severity, indicating a direct link between metabolic syndrome/MASH and HFpEF.

HOMAGE (Heart “Omics” in AGEing) research elucidated biomarkers’ predictive value for HFpEF, including in those with MASH. Elevated NT-proBNP and galectin-3 levels, indicative of myocardial stress and fibrosis, forecasted HFpEF development in metabolic disorder patients (Jacobs et al., 2014). Advanced imaging like cardiac magnetic resonance imaging (CMRI) revealed increased left ventricular mass, myocardial fibrosis, and impaired strain as HFpEF predictors in MASH patients (Wegermann et al., 2023). These findings underscore the intertwined coronary microvascular and diastolic dysfunctions’ role in MASH-related HFpEF pathogenesis, advocating for holistic cardiovascular assessment and management. Early interventions addressing cardiovascular risks in MASH may potentially curb HFpEF progression, emphasizing integrated care strategies for metabolic dysfunction’s hepatic and cardiac dimensions.

### MASH-associated cardiac structural and functional alterations

MASH is linked to cardiac structural and functional changes, extending its negative impact beyond hepatic dysfunction to significant cardiovascular implications. Ballestri *et al.* showed increased left ventricular mass and impaired diastolic function in MASLD patients (Ballestri et al., 2014), while van der Meer *et al.* found myocardial triglyceride accumulation associated with reduced left ventricular (LV) diastolic function in metabolic syndrome patients (van der Meer et al., 2008a; van der Meer et al., 2008b). Moreover, studies have reported changes such as increased diastolic posterior-wall thickness, LV mass, relative wall thickness and left atrial volume, (Chang et al., 2019; Ismaiel and Dumitrascu, 2019). Additionally, variations in ejection fraction, tissue Doppler imaging results, and the E/A ratio have been observed, indicating significant cardiac involvement in patients with MASLD (Petta et al., 2015; Lee et al., 2018). Magnetic resonance imaging (MRI) has further substantiated these findings, revealing noteworthy alterations in cardiac structure and function in individuals with MASLD, irrespective of the presence of clinically evident cardiac disease (Hallsworth et al., 2013). These findings highlight metabolic dysregulation’s direct effect on cardiac tissue, even in MASLD patients without major risk factors (Chang et al., 2019). Moreover, increased epicardial fat thickness has been linked to cardiac dysfunction via pro-inflammatory cytokine production (Petta et al., 2015). Further, the association between MASLD and a higher prevalence of atrial fibrillation highlights the potential for inflammatory milieu of MASH to foster electrical remodelling of the atria (Mantovani et al., 2019; Mantovani et al., 2022). Epidemiological studies also have drawn attention to the subtle yet persistent elevations in serum liver enzymes among MASLD patients, positioning these markers as predictive of the development of heart failure (Wannamethee et al., 2012; Wang et al., 2013). This correlation highlights the broader systemic consequences of liver pathology on cardiovascular health.

Compelling evidence links MASLD/MASH to a wide range of cardiovascular complications, identifying it as a critical risk factor for ASCVD, CADs, and alterations in LV function and structure. MASLD/MASH patients are notably at risk for carotid artery obstructions and exhibit significant changes in cardiac geometry and function, which, combined with impaired endothelial function and coronary plaque formation, significantly heighten cardiovascular event risks, including heart failure. The pathogenesis involves increased release of pro-inflammatory cytokines and LDL/VLDL from the liver, alongside risk factors like IR and dyslipidaemia. Cardiac assessments, particularly CFR measurements, are vital for identifying cardiovascular risks in MASLD/MASH patients. Despite well-established connections between MASH and cardiovascular disease, further research is crucial for understanding these complex interactions and improving management strategies, emphasizing the need for integrated cardiac and liver disease care to mitigate cardiovascular risks and enhance patient outcomes.

## Muscular complication in MASLD/MASH

Muscular tissue constitutes approximately 40% of total body weight and accounts for 50%–75% of all body proteins (Frontera and Ochala, 2015). It plays a pivotal role in metabolic processes, particularly in insulin-dependent glucose metabolism and fatty acid oxidation (Holecek, 2021). Functioning as an active endocrine entity, skeletal muscle influences inflammation regulation through the secretion of signalling molecules known as myokines (Hoffmann and Weigert, 2017). The integrity of muscle mass is crucial for maintaining muscle power, strength, and endurance, which collectively determine overall muscle performance. Hepatic steatosis disrupts the liver-muscle axis, initiating a detrimental cycle where liver disease impairs muscle protein synthesis and exacerbates metabolic imbalances, further fuelling MASLD and systemic inflammation (Bhanji et al., 2017a). This cycle is compounded by anabolic resistance in individuals with liver cirrhosis, a condition that diminishes skeletal muscle’s capacity to synthesize protein in response to nutrient intake, often leading to sarcopenia (Roman et al., 2014). Mechanistically, MASLD/MASH are associated with significant hormonal changes that impact muscle protein synthesis and degradation. Insulin-like growth factor-1 (IGF-1) levels are notably reduced in MASLD/MASH patients. IGF-1, produced mainly by the liver, plays a crucial role in muscle protein synthesis through the activation of the PI3K/Akt signaling pathway, which promotes muscle growth and inhibits protein degradation (Yoshida and Delafontaine, 2020). Conversely, myostatin, a negative regulator of muscle growth, is often elevated in MASLD/MASH (Meng et al., 2016). Myostatin inhibits muscle differentiation and protein synthesis by activating the SMAD2/3 signaling pathway, leading to muscle atrophy (Rodriguez et al., 2014). Increased myostatin levels are correlated with higher muscle protein degradation and reduced muscle mass in these patients (Yarasheski et al., 2002).

Additionally, alterations in adiponectin and leptin levels are observed in MASLD/MASH patients (Valenzuela-Vallejo et al., 2023; Venkatesh et al., 2024). Adiponectin, typically reduced in these conditions, has anti-inflammatory and insulin-sensitizing effects that support muscle protein synthesis (Yadav et al., 2013). Low adiponectin levels exacerbate muscle insulin resistance and protein breakdown. In contrast, leptin levels are often elevated and, while leptin promotes muscle protein synthesis in physiological conditions, chronic hyperleptinemia in MASLD/MASH can lead to leptin resistance, impairing its beneficial effects on muscle metabolism (Yadav et al., 2013; Polyzos et al., 2015). The hormonal imbalances in MASLD/MASH disrupt muscle protein synthesis and degradation, leading to sarcopenia and muscle wasting, highlighting the extensive impact on skeletal muscle metabolism and structural integrity.

### Sarcopenia in MASLD/MASH

Sarcopenia, characterized by the loss of muscle mass and function, emerges as a prevalent complication within MASLD, affecting up to 60% of patients with end-stage liver disease (ESLD) (Bhanji et al., 2017a; Bhanji et al., 2017b). This condition, beyond being a mere consequence of aging, is increasingly recognized as a progressive disease linked with higher risks of obesity, T2D, osteoporosis, CVDs, and cancer (Purnamasari et al., 2022; Yu et al., 2022; Damluji et al., 2023). The loss of muscle mass and strength in sarcopenia critically challenges physical performance and poses significant health risks, including increased disability, frailty, and mortality (Santilli et al., 2014; Lai et al., 2021). Sarcopenia shares pathophysiological pathways with MASLD, including metabolic dysfunction, hormonal imbalances, altered gut microbiome, and systemic inflammation (Lee et al., 2015; Petta et al., 2017; Wijarnpreecha et al., 2019). This multifaceted interaction contributes to a cycle where liver disease exacerbates muscle protein breakdown and inhibits synthesis, leading to decreased muscle mass and the onset of sarcopenia (Meyer et al., 2020). Factors such as gluconeogenesis, oxidative stress, mitochondrial dysfunction, and anabolic resistance play critical roles in this process, further complicated by the systemic effects of IR, adiposopathy, and hyperammonaemia (Walker, 2014; Holecek, 2021).

IR, a hallmark of MASH, disrupts normal insulin signalling, contributing to muscle loss, while adiposopathy promotes inflammatory pathways that aggravate sarcopenia through mechanisms like TNFα activation and myostatin release, inhibiting muscle protein synthesis (Merz and Thurmond, 2020; Sethi and Hotamisligil, 2021). Furthermore, NF-κB exacerbates sarcopenia by facilitating dyslipidaemia, while the accumulation of AT within skeletal muscles, referred to as myosteatosis, emerges as an additional outcome of adiposopathy (Gumucio et al., 2019; Joo and Kim, 2023). This condition notably impairs muscle strength and functionality, positioning myosteatosis as a critical indicator for both MASH and sarcopenia. MASH contributes to a reduction in muscle mass coupled with an escalation in fat accumulation, a phenomenon known as sarcopenic obesity (Gumucio et al., 2019; Nachit et al., 2021). In populations with chronic MASLD, statistics reveal that 43% exhibit sarcopenia, 26% are affected by sarcopenic obesity, and 52% display myosteatosis (Montano-Loza et al., 2016; Kang and Yoon, 2023). Additionally, vitamin D deficiency and low testosterone levels have been implicated in the pathology of sarcopenia within the MASLD context, suggesting potential therapeutic targets (Dhindsa et al., 2018; Zhang et al., 2022). Despite growing awareness, gaps in understanding the precise mechanisms and effective management strategies for sarcopenia in MASLD patients persist, necessitating further research to elucidate the contributions of MASH to sarcopenia and develop comprehensive treatment approaches.

### MASLD-associated inflammatory myopathies

Emerging evidence also points to the association of MASH with inflammatory myopathies, muscle disorders marked by skeletal muscle inflammation (Lundberg et al., 2021). Although traditionally linked to autoimmune conditions like dermatomyositis and polymyositis, MASH-related systemic inflammation has been implicated in muscle abnormalities’ development and progression (Bian et al., 2013; Chaudhry et al., 2019). Immune cell infiltration into skeletal muscles, accompanied by pro-inflammatory cytokine release, creates a local inflammatory milieu conducive to muscle damage, associated with MASH-related inflammatory “spill over” (Pillon et al., 2013). Inflammatory myopathies in MASLD may involve autoimmune responses, with autoantibodies and immune complexes contributing to muscle inflammation and damage (Lian et al., 2022). This process can lead to muscle fibre degeneration, manifesting as muscle weakness, fatigue, and impaired function, and complicating muscle repair and regeneration due to persistent inflammation (Mounier et al., 2013). Shared pathophysiological pathways between MASH and inflammatory myopathies involve chronic inflammation, immune dysregulation, and oxidative stress, contributing to muscle damage and inflammation. While the association between MASH and inflammatory myopathies is recognized, further research is warranted to comprehend underlying mechanisms and clinical implications. Identifying and addressing muscle inflammation in MASH may have therapeutic benefits by reducing systemic inflammation and immune dysregulation, potentially improving muscle function and overall disease outcomes.

## Renal complications in MASLD/MASH

Renal complications, particularly chronic kidney disease (CKD), are integral to the systemic impact of MASLD/MASH, underscoring the complex interplay of metabolic disorders (Byrne and Targher, 2020; Cao et al., 2021; Umbro et al., 2021). CKD, characterized by progressive renal function loss, involves waste product accumulation and disruptions in fluid and electrolyte balances, often evidenced by biomarkers indicating kidney damage or reduced glomerular filtration rate (GFR) (Byrne, 2013; Byrne and Targher, 2020). In the United States, the prevalence of individuals necessitating renal replacement therapy exceeds 400,000, with projections indicating a surge to 2.2 million by 2030, highlighting the escalating burden of kidney disease (Marcuccilli and Chonchol, 2016). Emerging clinical research has identified MASLD/MASH as significant independent predictors for both the onset and progression of CKD, suggesting a profound link between hepatic steatosis and renal dysfunction (Byrne, 2013; Byrne and Targher, 2020). These studies elucidate that the severity of liver disease, characterized by fat accumulation and inflammation in the liver, correlates with the risk of developing kidney complications, positioning MASLD/MASH within the broader constellation of metabolic syndrome-related conditions that adversely affect renal health.

The pathophysiological bridges between MASLD/MASH and CKD are multifaceted, encompassing impaired antioxidant defences, persistent low-grade systemic inflammation, activation of the renin-angiotensin system, and aberrant lipid metabolism (Byrne and Targher, 2020; Bilson et al., 2023; Li et al., 2023). These factors collectively contribute to a milieu conducive to renal injury. Specifically, the role of pro-inflammatory, pro-fibrogenic, and anti-fibrinolytic mediators such as fetuin-A, fibroblast growth factor (FGF)-21, TNF-α, transforming growth factor (TGF)-β, and plasminogen activator inhibitor-1 (PAI-1), has been implicated in promoting kidney damage through mechanisms that include the exacerbation of inflammation and fibrosis within the renal tissue (Musso et al., 2014; Byrne and Targher, 2020). Additionally, liver-derived metabolites, including uremic toxins, play a significant role in the pathogenesis and progression of CKD. Uremic toxins such as indoxyl sulfate (IS), p-cresyl sulfate (PCS), and trimethylamine N-oxide (TMAO) are primarily generated in the liver through the metabolism of dietary components by gut microbiota, followed by hepatic processing (Zhen et al., 2023). These metabolites are recognized for their nephrotoxic effects and their contribution to CKD progression (Castillo-Rodriguez et al., 2018). Indoxyl sulfate, a protein-bound uremic toxin derived from the metabolism of tryptophan, is one of the most studied nephrotoxic metabolites. Once produced in the liver, IS poorly eliminated by the kidneys in CKD patients, leading to its accumulation. Elevated IS levels induce oxidative stress and inflammation in renal tubular cells, promoting fibrosis and accelerating the decline in renal function (Barreto et al., 2009). Moreover, IS was also found to be increased in MASLD patients (Hotamisligil, 2006; Ribeiro et al., 2023). Mechanistically, IS activates the aryl hydrocarbon receptor (AhR) pathway and induces the expression of TGF-β and pro-inflammatory cytokines, thereby exacerbating renal injury (Delgado-Marin et al., 2024). Similarly, PCS, a metabolite of tyrosine, has been implicated in CKD progression through mechanisms akin to those of IS. PCS induces endothelial dysfunction and increases vascular permeability, contributing to renal damage and cardiovascular complications commonly seen in CKD patients (Meijers et al., 2010). Additionally, PCS has been shown to inhibit the proliferation and repair of renal epithelial cells, further impairing renal function (Han et al., 2015).

TMAO, another liver-derived metabolite, originates from the hepatic oxidation of trimethylamine, a product of gut microbial metabolism of choline, phosphatidylcholine, and carnitine. Elevated TMAO levels are associated with adverse renal outcomes, including glomerular sclerosis and interstitial fibrosis. TMAO enhances renal oxidative stress and inflammatory responses, contributing to the progression of CKD (Tang et al., 2015). Moreover, TMAO has been linked to the upregulation of pro-fibrotic and pro-inflammatory genes, thereby exacerbating renal fibrosis and dysfunction (Zhang et al., 2020). These liver-derived metabolites, by inducing oxidative stress, inflammation, and fibrosis, significantly contribute to the nephrotoxic milieu in CKD.

Despite the accumulating evidence of a link between MASH and CKD, establishing a definitive causal relationship remains challenging. The complexity of metabolic syndrome, with its array of cardiovascular, hepatic, and renal manifestations, necessitates a holistic understanding of these interconnected systems. Further research, employing longitudinal studies and advanced biomolecular techniques, is required to dissect the intricate mechanisms by which MASLD/MASH contributes to the development and progression of CKD. Such insights will be crucial for devising targeted therapeutic interventions aimed at mitigating renal complications in patients with metabolic liver disease, thereby addressing an important component of the morbidity and mortality associated with this condition.

## Pathophysiological links between MASLD/MASH and extrahepatic complications

The pathophysiological interrelation between MAFLD or MASH and its extraneous manifestations, notably CVDs, CKD and muscular anomalies, constitutes a multidimensional domain marked by inflammation and oxidative stress, perturbations in gut microbiota composition, and dyslipidaemia. Each component intricately contributes to this complex network of pathogenesis.

### Inflammation and fibrosis

Metabolic inflammation in MASH is marked by a systemic, low-grade inflammatory response triggered by factors like high-fat diets. This systemic inflammation is facilitated by the infiltration of inflammatory cells such as macrophages and lymphocytes into the liver, releasing pro-inflammatory cytokines that contribute to liver damage (Buzzetti et al., 2016; Bhanji et al., 2017a; Boesch et al., 2023). Kupffer cells, the liver’s resident macrophages, are central to orchestrating liver inflammation (Jensen et al., 2018; Cusi et al., 2024). The crosstalk between hepatocytes and immune cells, mediated by inflammasomes, plays a crucial role in this process (Fontes-Cal et al., 2021; Duan et al., 2022). Inflammation leads to fibrosis, an abnormal wound healing response characterized by the deposition of extracellular matrix proteins and formation of scar tissue, potentially progressing to cirrhosis (Iwakiri and Trebicka, 2021; Zhao X. et al., 2022). Cirrhosis increases resistance in hepatic vasculature, leading to portal hypertension, collateral vessel formation, and increased cardiac workload, potentially resulting in heart failure (Gonzalez et al., 2020; Iwakiri and Trebicka, 2021; Umbro et al., 2021).

### Insulin resistance

A key feature of MASLD and MASH, IR disrupts glucose homeostasis, promoting fat accumulation in the liver and progressing from simple steatosis to MASH and potentially to cirrhosis and liver failure (Llovet et al., 2023; Cusi et al., 2024). IR fosters inflammation and oxidative stress, contributing to liver cell injury and disease progression (Loomba et al., 2021; Muzurovic et al., 2022; Njoku et al., 2022). The complex mechanisms underlying IRinvolve genetic factors, obesity, inflammation, lipotoxicity, mitochondrial dysfunction, and hormonal imbalances (Powell et al., 2021; Salah et al., 2021; Tripathi et al., 2022; Wong et al., 2023).

### Gut microbiota

Dysbiosis, or the imbalance of gut microbiota, has been increasingly recognized for its role in MASH and CVD (Brody, 2020; Violi et al., 2023). Increased gut permeability, or “leaky gut,” allows toxins to reach the liver, exacerbating inflammation and damage (Brody, 2020; Song and Zhang, 2022; Violi et al., 2023). Endotoxins from gut bacteria, such as lipopolysaccharides (LPS), stimulate inflammation, contributing to liver injury (Solanki et al., 2023; Violi et al., 2023). Alterations in gut microbiota also affect bile acid metabolism, influencing liver inflammation and metabolism (Xiang et al., 2022). Therapeutic modulation of gut microbiota through probiotics, prebiotics, dietary changes, short-chain fatty acids (SCFAs) or faecal microbiota transplantation has shown promise in improving MASH outcomes (Philips et al., 2020; Nogal et al., 2021; Xiang et al., 2022).

### Dyslipidaemia

Characterized by abnormal levels of blood lipids, dyslipidaemia is a crucial risk factor for CVD, ASCVD, CAD and CKD (Linton et al., 2000; Stasi et al., 2022). High LDL cholesterol and triglycerides contribute to atherosclerosis, while low HDL cholesterol is linked to increased cardiovascular risk (Linton et al., 2000). Dyslipidaemia not only contributes to MASH development but also exacerbates the condition, necessitating comprehensive management to mitigate cardiovascular and liver damage risks (Mendez-Sanchez et al., 2020).

### Genetics

Genetic predispositions play a significant role in the susceptibility to MASLD and its progression (Younossi et al., 2023). Variants in genes related to lipid metabolism (e.g., PNPLA3, TM6SF2), inflammation, oxidative stress, and fibrosis have been implicated in increasing the risk of developing MASLD (Anstee et al., 2020; Sveinbjornsson et al., 2022; Xie et al., 2023). These genetic factors affect liver fat processing and storage, lipid metabolism, and the inflammatory response, underscoring the genetic complexity of MASLD and its systemic manifestations.

### Hepatokines

Unlike liver-derived metabolites, hepatokines are the molecular transducers with hormone-like activities (Seo et al., 2021). These are central to the inter-organ communication that regulates metabolic homeostasis (Stefan et al., 2023). These molecules are implicated in a variety of physiological processes including glucose and lipid metabolism, inflammation, and energy homeostasis (Jensen-Cody and Potthoff, 2021; Khan et al., 2022; Stefan et al., 2023). As metabolic regulators, hepatokines provide a mechanistic link between liver function and metabolic disorders such as diabetes, obesity, CVDs, CKD, and sarcopenia. Some previous reports have reviewed these hepatokines excellently (de Oliveira Dos Santos et al., 2021; Jensen-Cody and Potthoff, 2021; Khan et al., 2022; Berezin et al., 2023; Stefan et al., 2023). Few of the important hepatokines are summarized in Table 1 with their target organs, and their physiological roles. Mechanistically, hepatokines exert their effects through autocrine, paracrine, or endocrine mechanisms, influencing not only the liver itself but also distant organs. These effects can be either beneficial, such as improving insulin sensitivity, or detrimental, such as promoting IRand inflammation depending on the pathological state of the organism. For instance, fibroblast growth factor 21 (FGF21), which is a stellar hepatokine among others, modulates glucose and lipid metabolism in cardiac and muscle tissues (Jimenez et al., 2018; Zhang et al., 2019). The extensive influence of hepatokines on multiple organ systems highlights their potential as targets for therapeutic intervention in metabolic diseases. Understanding the specific roles of hepatokines not only aids in translating the pathophysiology of metabolic disorders but also opens new ways for treatment, such as the use of recombinant FGF21 or its analogs for CVDs, CKD and muscle disorders during MASH (Sunaga et al., 2019). Future research focusing on the interaction between hepatokines and their receptors may provide novel insights into their mechanistic pathways and therapeutic potential.

**TABLE 1:** Table summarizing hepatokines, detailing their primary target organs and functions.

Hepatokine	Target organs	Functions	References
FGF21	Muscle, AT, pancreas, brain	Enhances insulin sensitivity, modulates lipid metabolism, regulates energy expenditure	Liu et al. (2015), Xie and Leung (2017)
ANGPTL3	Liver, AT	Inhibits lipoprotein lipase, regulates lipid metabolism	Santulli (2014)
ANGPTL6	Muscle, cardiovascular system	Modulates glucose metabolism, promotes angiogenesis	Santulli (2014)
Selenoprotein P	Muscle, pancreas, brain	Antioxidant, regulates glucose and insulin metabolism	Misu et al. (2010)
Hepcidin	Intestine, liver, spleen	Regulates iron metabolism, modulates inflammatory responses	Ganz and Nemeth (2012)
Betatrophin	Pancreas, AT	Regulates β-cell proliferation, impacts lipid metabolism	Raghow (2013)
Fetuin-A	AT, immune system	Induces inflammation, modulates insulin signaling	Peter et al. (2018)
RBP4	AT, muscle	Modulates insulin resistance, regulates glucose homeostasis	Graham et al. (2006)
FGF19	Gallbladder, intestine, liver	Regulates bile acid synthesis, modulates energy expenditure	Kir et al. (2011)
Leptin	Brain, AT	Regulates appetite, energy balance, insulin sensitivity	Li (2011)
Adropin	Brain, cardiovascular system	Regulates energy homeostasis and vascular function	Kumar et al. (2008)
Follistatin	Muscle, liver, AT	Muscle growth, modulation of metabolism, suppression of myostatin	Lee and McPherron (1999)
DPP4	AT, liver, immune system	Enzymatic activity influencing glucose metabolism, inflammation	Deacon (2011)
FGF23	Bone, kidneys	Regulates phosphate metabolism, vitamin D levels	Shimada et al. (2004)
GDF15	Brain, AT	Regulates appetite, inflammatory responses	Plomgaard et al. (2022)
Osteopontin	Immune system, bone	Regulates immune responses, bone remodeling	Scheller et al. (2011)
Angptl8	AT, liver	Regulates lipid metabolism, modulates lipoprotein lipase activity	Ebert et al. (2014), Perdomo et al. (2021)

The association between MASH and its extrahepatic manifestations, especially CVDs and CKD, is reinforced by complex pathophysiological mechanisms. While inflammation, fibrosis, IR, dysbiosis, dyslipidaemia, and genetic factors each contribute to the disease process, their interplay exacerbates the systemic impact of MASH. Understanding these mechanisms is crucial for developing targeted therapeutic strategies to manage MASLD and its broad spectrum of complications. Further research is needed to elucidate these complex interactions fully and identify effective interventions for patients with MASH.

## Multidisciplinary and innovative therapeutic strategies for managing MASH and associated extrahepatic manifestations

**FIGURE 3:**
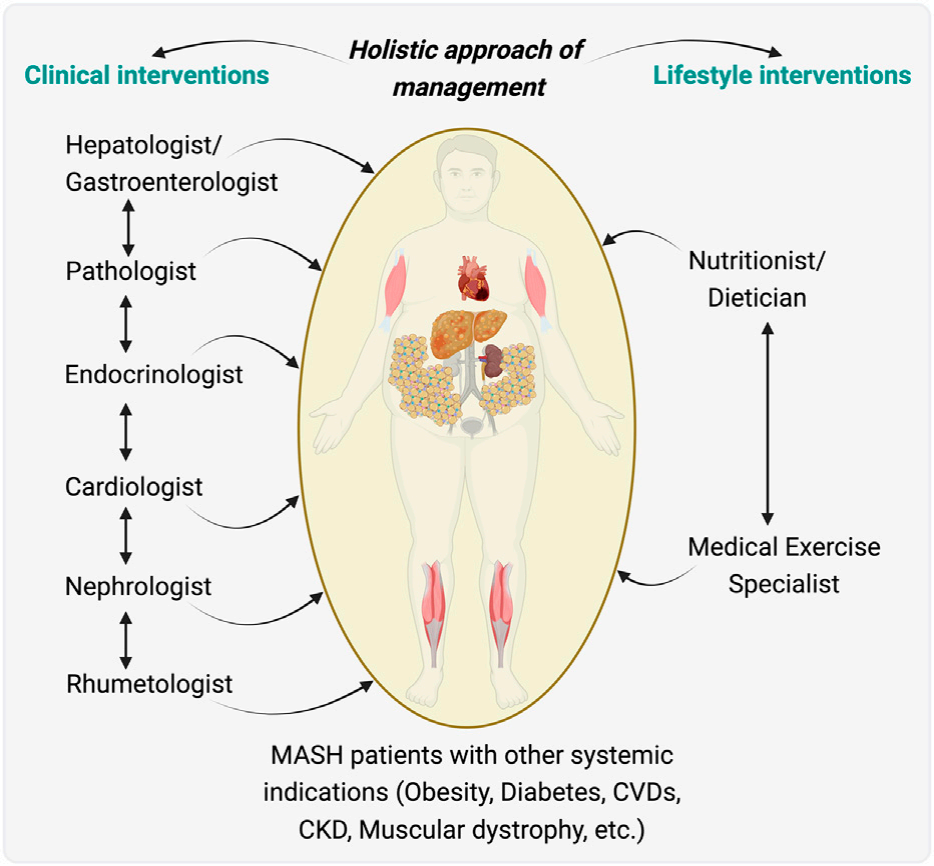
An Integrated holistic care model for managing patients with metabolic associated steatohepatitis (MASH) and other MASH-associated effects. Illustration proposed an integrated care model for patients with MASH who also have systemic indications such as obesity, diabetes, cardiovascular diseases (CVDs), chronic kidney disease (CKD), and muscular dystrophy. Clinical interventions proposed are outlined, showing a flow of care from a hepatologist/gastroenterologist to a team including a pathologist, endocrinologist, cardiologist, nephrologist, and rheumatologist, indicating the need for collaborative medical management across different organ systems affected during/by MASH. Lifestyle interventions are highlighted, with a nutritionist/dietitian and a medical exercise specialist playing key roles in managing the patient’s nutrition and physical activity, essential for the overall treatment strategy. This model highlights the importance of a multidisciplinary integrative team working in concert to provide comprehensive care that addresses the multifaceted aspects of MASH and its related systemic diseases. The illustration was made on BioRender.com.

The management of MASH and its associated systemic/extrahepatic manifestations, including CVDs, sarcopenia, and CKDs, demands a holistic and multidisciplinary approach (Figure 3). Collaboration among hepatologists, endocrinologists, dietitians, and exercise specialists are essential to address the multifaceted nature of the disease comprehensively. Tailoring treatment plans to individual patient profiles, considering disease severity and comorbidities, is paramount for optimizing health outcomes. Lifestyle interventions, focusing on diet, exercise, and weight management, serve as the cornerstone of MASH treatment (Machado, 2021; Semmler et al., 2021; Viveiros, 2021), aiming to mitigate metabolic derangements, reduce hepatic fat accumulation, enhance insulin sensitivity, and alleviate inflammation and oxidative stress. For example, caloric restriction and adherence to a Mediterranean-style diet have shown promise in diminishing hepatic fat and improving liver health, while aerobic and resistance training exercises enhance insulin sensitivity and support weight loss (Hallsworth et al., 2011; Anania et al., 2018; Ristic-Medic et al., 2020). Addressing micronutrient imbalances, such as deficiencies in vitamins B12, folate, and potentially beneficial supplements like vitamin E and omega-3 fatty acids, may offer therapeutic benefits by modulating hyperhomocysteinemia, inflammation, oxidative stress, and liver fibrosis (Calder, 2010; Koplay et al., 2011; Ryan et al., 2013; Mahamid et al., 2018; Musa-Veloso et al., 2018; Zaric et al., 2019; Levy et al., 2021; Tripathi et al., 2022).

Emerging therapeutic targets and pharmacological interventions represent significant areas of active research. The exploration of thyromimetics, which mimic thyroid hormone actions to regulate metabolism, demonstrates potential in ameliorating MASH-related liver damage, inflammation, and fibrosis, as evidenced by clinical trials with agents like resmetirom (Sinha et al., 2020). Similarly, agents targeting metabolic pathways, such as GLP-1 receptor agonists and PPARα agonists, are under investigation for their capacity to correct metabolic abnormalities integral to MASH pathophysiology (Zhu et al., 2021; Zhang et al., 2023). Moreover, the complex interplay between MASH and gut microbiota suggests that modulating the gut microbiome through probiotics, prebiotics, or faecal microbiota transplantation could offer a novel avenue for treatment, addressing dysbiosis and its contributions to liver inflammation and damage (Xiang et al., 2022; Solanki et al., 2023).

Given MASH’s association with metabolic disturbances, combination therapies targeting various aspects of the disease process, ranging from inflammation and fibrosis to metabolic dysfunctions, are likely to emerge as a promising strategy. The synergy between pharmacological agents with complementary mechanisms of action could enhance treatment efficacy, potentially offering a more comprehensive approach to managing MASH and its complications. However, the path to optimizing MASH management extends beyond current therapeutic modalities. Future research directions include the identification of specific molecular and genetic markers to guide personalized treatment strategies, the utilization of non-invasive imaging techniques for liver assessment, and the exploration of novel therapeutic targets. Such advancements are anticipated to refine therapeutic interventions, improve prognostication, and ultimately enhance the quality of life for patients with MASH.

## Conclusion

The management of MASH necessitates a comprehensive, personalized strategy that encompasses lifestyle interventions, pharmacological measures, and emerging therapeutic agents to address both hepatic and systemic manifestations. Central to this approach is the recognition of MASH as a systemic disorder with significant impacts on cardiovascular health, skeletal muscle function, and renal integrity. The interconnections between MASH and its extrahepatic effects underline the importance of a multidisciplinary care model, emphasizing early detection, precise risk assessment, and tailored treatment plans that mitigate the progression of associated conditions. Key lifestyle modifications such as dietary adjustments, increased physical activity, and weight management form the cornerstone of MASH management, targeting causal metabolic dysfunctions including IR and dyslipidaemia. Additionally, addressing chronic inflammation and oxidative stress through targeted pharmacological interventions is critical for ameliorating the broader health impacts of MASH. As research advances, the potential for novel therapies targeting specific pathophysiological pathways offers hope for more effective and individualized treatments, aiming to improve overall patient outcomes and reduce the burden of complex MASLD and MASH. Future research directions should concentrate on unveiling new therapeutic targets, adopting precision medicine strategies, and understanding the long-term effects of MASH on organ health to optimize care for affected individuals.

## Abbreviations

AT, Adipose tissue; ALT, Alanine aminotransferase; ASCVD, Atherosclerotic cardiovascular diseases; BMI, Body mass index; CRP, C-reactive protein; CMRI, Cardiac magnetic resonance imaging; CVDs, Cardiovascular diseases; CIMT, Carotid intima-media thickness; CKDs, Chronic kidney diseases; CT, Computed tomography; CAD, Coronary artery diseases; CFR, Coronary flow reserve; ESLD, End-stage liver disease; ER, Endoplasmic reticulum; FXR, Farnesoid X Receptor; FA, Fatty acids; HFpEF, Heart failure with preserved ejection fraction; IR, Insulin resistance; LV, Left ventricular; LDs, Lipid droplets; MASH, Metabolic dysfunction associated steatohepatitis; NASH, Non-alcoholic steatohepatitis; NF-κB, Nuclear factor kappa-light-chain-enhancer of activated B cells; PPAR, Peroxisome proliferator-activated receptor; PAI-1, Plasminogen activator inhibitor-1; SCFAs, Short-chain fatty acids; TGF-β, Transforming growth factor; TNFα, Tumor necrosis factor α; T2D, Type 2 diabetes mellitus; VLDL, Very-low-density lipoprotein.
